# What is public trust in national electronic health record systems? A scoping review of qualitative research studies from 1995 to 2021

**DOI:** 10.1177/20552076241228024

**Published:** 2024-01-28

**Authors:** Kimon Papadopoulos, Viktor von Wyl, Felix Gille

**Affiliations:** 1 Digital Society Initiative (DSI), 30841 University of Zürich, Zurich, Switzerland; 2 Institute for Implementation Science in Health Care (IfIS), 27217 University of Zürich, Zurich, Switzerland

**Keywords:** Public trust, digital health, electronic health records, patient data, scoping review, qualitative research

## Abstract

**Objective:**

Public trust in national electronic health record systems is essential for the successful implementation within a healthcare system. Research investigating public trust in electronic health records is limited, leading to a lack of conceptual clarity. In response, the objective of this study is to gain a clearer understanding on the conceptualizations of public trust in electronic health records, which can support the implementation of national electronic health record systems.

**Methods:**

Guided by the PRISMA-ScR checklist, a scoping review of 27 qualitative studies on public trust in electronic health records found between January 2022 and June 2022 was conducted using an inclusive search method. In an iterative process, conceptual themes were derived describing the promoters and outcomes of public trust in electronic health records.

**Results:**

Five major conceptual themes with 15 sub-themes were present across the literature. Comprehension, autonomy, and data protection promote public trust in electronic health record; while personal and system benefits are the outcomes once public trust in electronic health records exists. Additional findings highlight the pivotal role of healthcare actors for the public trust building process.

**Conclusions:**

The results underscore comprehension, autonomy, and data protection as important themes that help ascertain and solidify public trust in electronic health records. As well, health system actors have the capacity to promote or hinder national electronic health record implementation, depending on their actions and how the public perceives those actions. The findings can assist researchers, policymakers, and other health system actors in attaining a better understanding of the intricacies of public trust in electronic health records.

## Introduction

Electronic health records (EHRs) are health records that contain aspects of an individual's healthcare history in a digital format. EHRs allow this data to be stored and shared with efficiency and security. They are also a valuable component for supplying and communicating health data within a national healthcare system.^1^ EHRs are an instrumental asset in the enhancement of quality of care, the reduction of medical costs and medical errors, the advancement of healthcare research, support in health emergencies, and to the overall efficiency of the healthcare system.^2–4^ Despite the proven capabilities of EHRs within healthcare systems, successful implementation in many European healthcare systems remains unrealized.^5,6^ Next to legal, technical, and economic challenges, the lack of public trust in EHRs is a key inhibitor.^6–9^

Public trust can be described as the expectation of the general public that institutions, organizations, and/or individuals responsible for managing, supervising, and/or providing services to act in the public's interests.^10^ It is bound to a feeling of integrity, reliability, and competence of these entities and of optimism that they are acting with the public’s best interests at heart, as trust cannot operate without a belief of benevolence.^10^ This is significant as government officials require adequate trust in the healthcare system to promote uptake and facilitation of public health and healthcare interventions, such as EHRs.

In the context of EHRs, public trust in both the healthcare system and its actors to keep health information secure, autonomous, and used benevolently is crucial to support their implementation of EHRs and the use of data. Without such trust, people are less likely to consent to sharing their health data through an EHR.^7^ Yet, fostering public trust in EHRs remains challenging, as there is a lack of a clear consensus on the definition of public trust in EHRs. This lack of consensus is especially prominent within the varying healthcare systems across the European region.^11^ Reaching clarity on what public trust means in the context of EHRs is a necessary step in constructing effective policy and health system actions that advances the successful implementation of EHRs into a country's healthcare system. However, there are obstacles to reaching a consistent understanding of the concept of public trust in EHRs, as qualitative research into both trust and digital health interventions (in our case, EHRs) is still relatively nascent. The fact of the matter is, if we do not agree on what public trust in an EHR system is, it will be difficult to develop targeted health policy actions to promote public trust and effective measures to better understand levels of public trust.

To contribute to the closure of this conceptual gap, this review seeks to synthesize the conceptual understandings about public trust in national EHR systems throughout the current qualitative literature up to 2021. The findings of this study will be informative on what public trust in EHRs means and will help to inform future research on public trust in EHRs across different countries, cultures, and languages. Moreover, the findings can guide the development of trust building health policy, inform academic and health policy debate, and serve as a basis for studies aiming to measure public trust in EHRs.

## Methods

### Search strategy and study selection

We conducted a scoping review of qualitative studies that investigated trust in EHRs with patients (members of the public who are receiving medical care) and/or members of the public as participants.^12,13^ The review was guided by the PRISMA-ScR checklist.^14^ The review focused on original research articles presenting qualitative studies to facilitate conceptual development and understanding, as qualitative research is more effective quantitative survey analyses on public trust in EHRs in discovering the nuances inherent in researching trust and allows for a clearer picture on what public trust in EHRs constitutes.^15^

Utilizing advice given by a Library Subject Specialist from the University Zurich Library, we utilized an inclusive search string across all selected databases to maximize results. The MeSH terms and search string used for this review were “trust” and “electronic health record.” This open search string was chosen as more narrowed searches produced limited results. The databases used for our search were: CINAHL, Cochrane Library, PsychINFO, PubMed, and ScienceDirect. These were selected due to their large article base that entailed research articles covering qualitative studies.

The articles needed to meet the inclusion criteria: qualitative interview studies with patients and/or members of the public as participants that investigated trust in EHRs. Only studies in English that were published before June 2022 were included.

### Data extraction and synthesis

**Figure 1.**
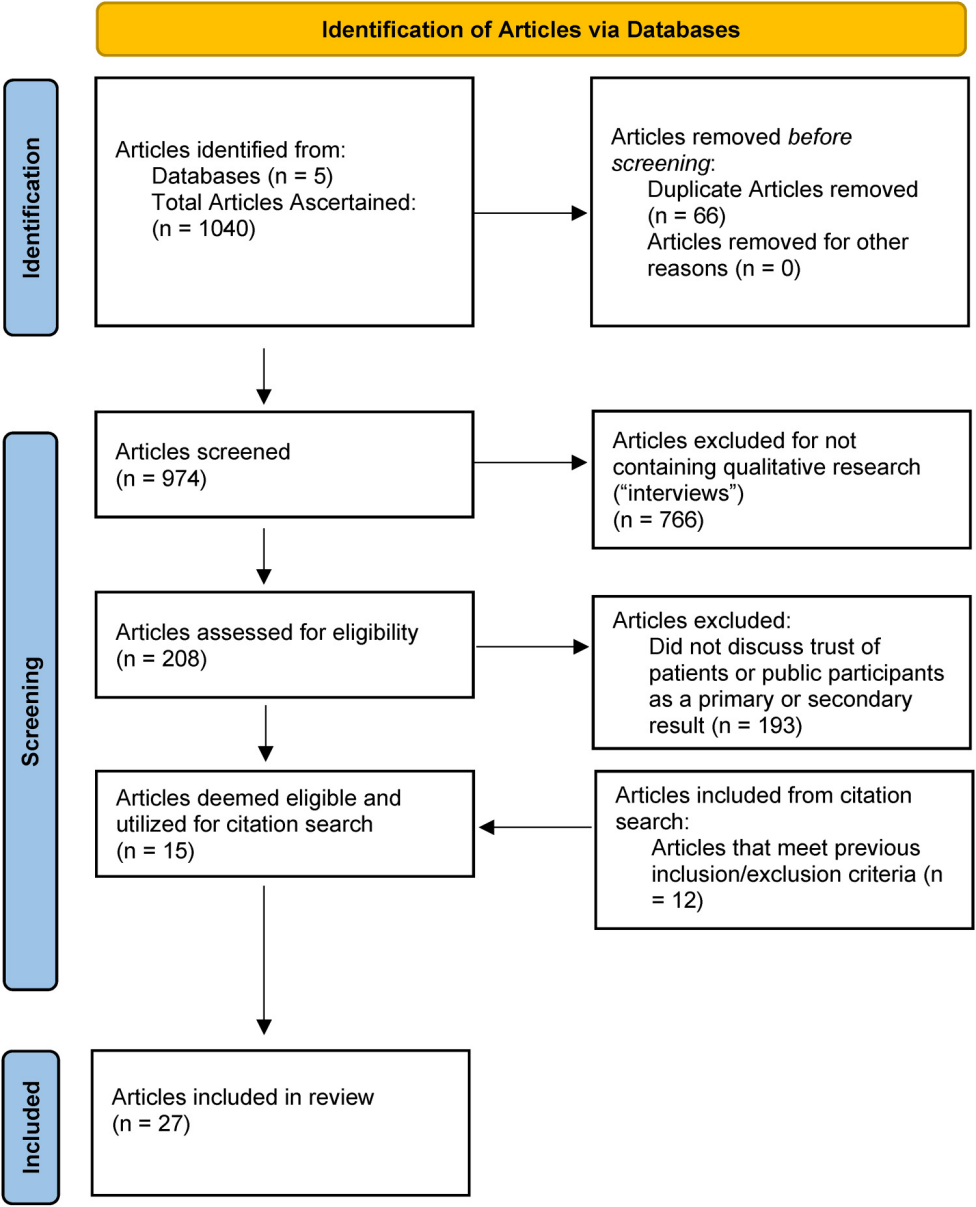
Literature screening process.

**Figure 2.**
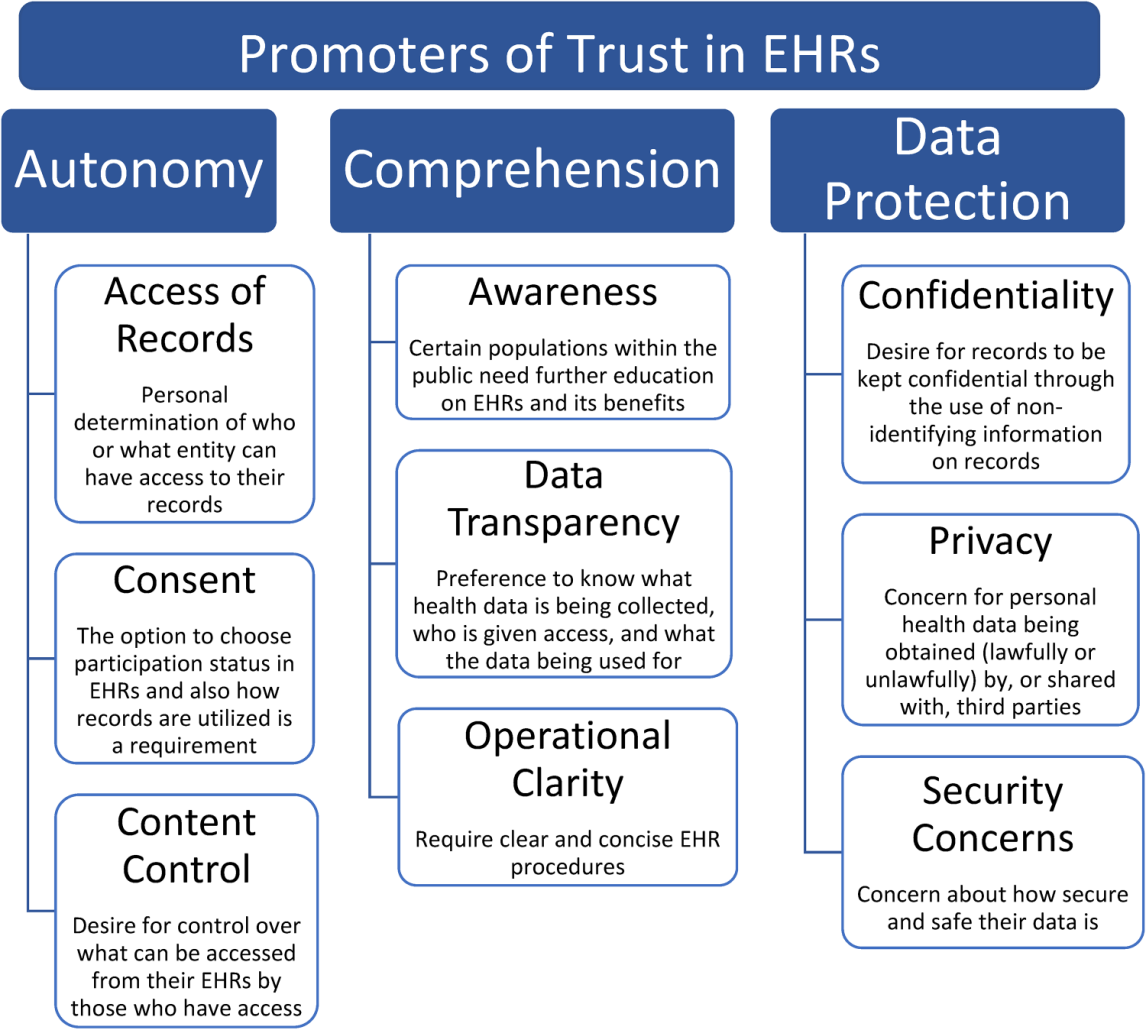
Promoters of public trust in EHRs.

**Figure 3.**
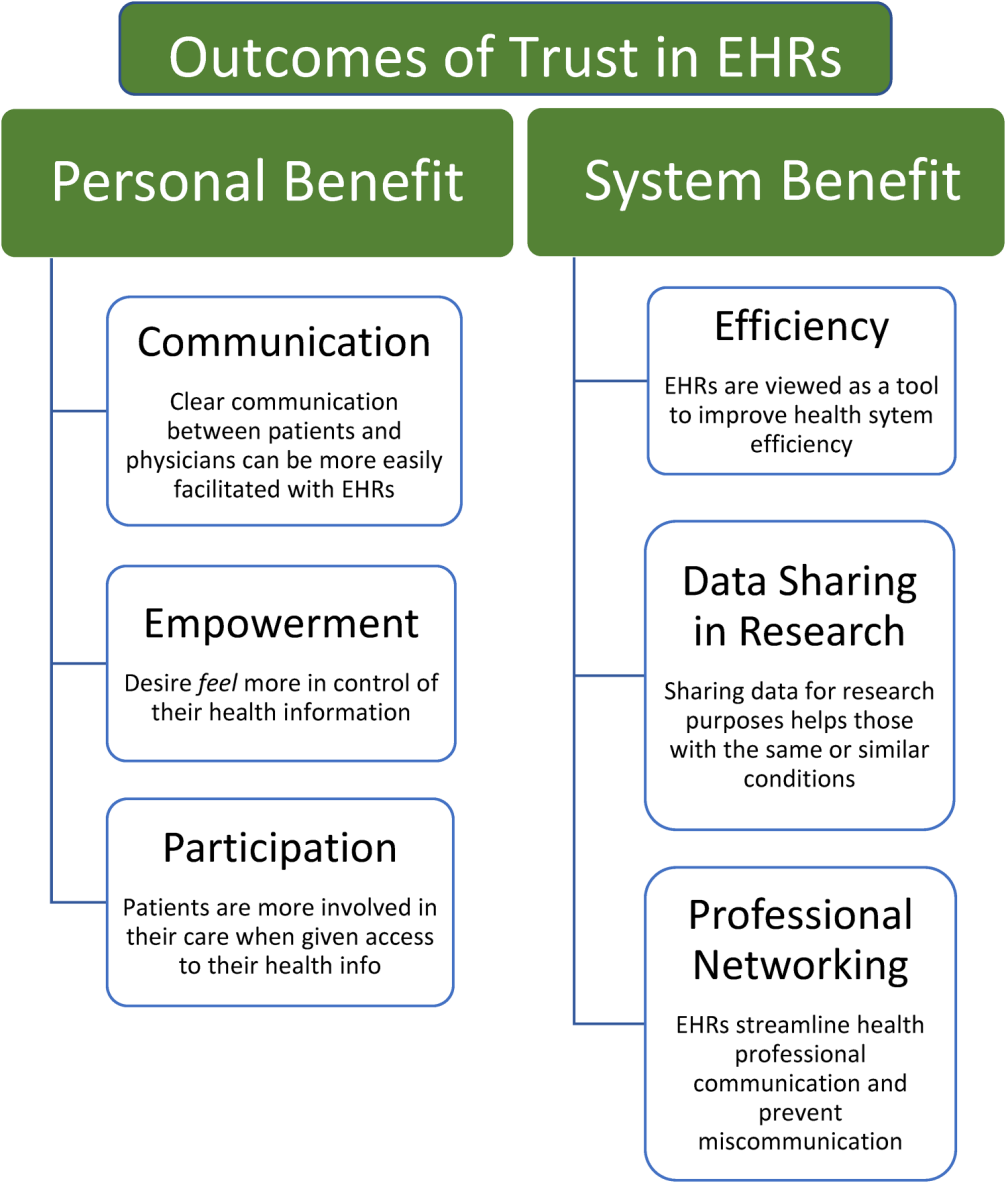
Outcomes of public trust in EHRs.

Our search revealed 1040 articles, 66 of which were duplicates (Figure 1). The remaining 974 articles were further narrowed by using a title, abstract, and keyword screening to determine the articles that contained reference to “interviews” to identify which ones presented qualitative research methods. Two hundred and eight articles were identified, with the remaining 766 being excluded. The 208 included articles were then screened by KP and FG and were sieved for qualitative studies interviewed patients or members of the public. Our final study sample consisted of 15 articles. From the 15 articles, we conducted a citation search to determine if more literature on the topic of interest could be found. A further 12 articles met the inclusion criteria and were included in our study (Figure 1). These 27 articles were then inductively analyzed in an iterative process by KP to determine common themes emerging in the literature, with a review of KPs analysis and a following reanalysis conducted by FG.^16^ Any discrepancies between the two analyses were discussed by both KP and FG and rectified in a collaborative dialogue between both parties. All analyses were conducted using the qualitative analysis software MAXQDA. The MAXQDA codes were developed utilizing established inductive content analysis and are presented in Figure A (Appendix).^16^ Developing themes throughout the literature were identified, examined, and summarized into theme trees based upon conceptual correlation development and work previously done by FG (Figures 2 and 3).^11^

## Results

The 27 articles were published by researchers from Europe and the United States, with one exception, which was conducted in Canada. 55.6% (*n*  =  15) of studies were conducted in Europe, while 40.7% (*n*  =  11) were conducted in the United States, with 3.7% (*n*  =  1) having been conducted in Canada.

A total of 15 sub-themes were identified under five key themes: comprehension, autonomy, data protection, personal benefit, and system benefit (Figures 2 and 3). The 15 sub-themes were presented under 5 “major themes” in alphabetical order to create “theme trees.” The theme trees offer a clearer understanding of the interplay between the different themes, i.e., how some have thematic interlinks with one another but are also separate to others. The major themes were divided into two categories after analysis: (a) the blue major themes relate to the concepts that potentially produce public trust in an EHR system, and (b) the green major themes relate to the public-perceived benefits of EHRs once significant trust levels are achieved among the public.

While searching for common themes within the literature during the course of our thematic analysis and discovery, we identified an additional finding that was not explicitly mentioned by the literature pointing towards the key role of health system actors for the trust relationship between the public and EHRs. These actors play pivotal roles in how the public comes to trust EHRs in the health system, with each actor having the ability to promote or hinder EHR implementation, with the effect of their influence being subject to their actions and how the public perceives those actions. Despite the fact that they are not explicitly defined or discussed within the reviewed literature, they were included in our analysis as the presence and influence of these actors are present in much of the literature and are an important aspect of trust in EHRs.

### Autonomy

Autonomy refers to the desire from the public who use EHRs to have the “independent authority” of determining whether they participate in EHRs, who is given access to their records, and what specific information is shared with those who have access. Without autonomy, the public have a hard time trusting the use of EHRs.

#### Access of records

Due to concerns over who can get access to their sensitive health data, people more likely trust EHRs when they are the ones who have oversight over who and how many people have access to their records.^17–19^ People are willing to have medical professionals who are directly treating them have access. However, they request that the medical professional have a patients consent first, and that administration and secretarial staff, along with doctors who are not involved in their care, are not be given access.^20,21^ In addition, it has been found that public trust is built when the patients themselves are given the ability to determine who can access their records, whether it be a family member, a third-party entity (such as paramedics and other first responders in an emergency situation), and medical professionals of their choosing.^22,23^

#### Consent

A key aspect of a person's autonomy in relation to EHRs is the consent. People want to be asked for their consent before anything is done with their health data, whether it is to collect their data into an EHR, or to even to use their data for medical use or research purposes.^17,18,21,24–26^ There is also an observed negative perception on the use of an “opt-out” system of EHR participation. Participants across multiple studies took issue with the “implied permission” that an “opt-out” approach brought. This has led to a seeming overwhelming support for an “opt-in” approach, where participants are more actively asked for their consent to participate.^18,24–26^

#### Content control

When a person gives access of their private EHR to another individual or entity, many wish to have only certain parts of their record made available.^27^ Additionally, they want to be the ones in control over these redactions.^22^ Specifically, some individuals want to negotiate with doctors themselves over what should and should not be in the EHR in the first place.^20^ Others suggest a categorical approach, where only certain people are allowed to view certain parts of their health data, except for certain medical professionals who are allowed full access.^24,28^ This is not to be confused with consent, which deals with the opening ones data into the health system, control is more about controlling the flow of health within the EHR system once it has already entered. Overall, with greater control over their EHRs, people feel higher levels of trust towards the healthcare system that deploys the EHRs to the public.^29^

### Comprehension

According to the public, gaining more robust comprehension on what EHRs are, how EHRs work, and why and how their data is being collected and used, is an important requirement. From our results, three distinct themes contribute to the comprehension of EHR operations that enable public trust in EHRs: awareness, data transparency, and operational clarity.

#### Awareness

The literature highlighted how members of the public are often times unaware of what EHRs are, what they are used for, why they are used, and/or how they can be beneficial to health.^21,25,30–32^ In one instance, participants in Germany and Austria were asked how familiar they were with the term “EHR.” Only 30.5% and 32.5% of Austrian and German respondents, respectively, reported that they were familiar with the term. However, those who said they were familiar were then asked to describe the concept of an EHR in their own words. Fifty percent of Austrian participants and 56% of German participants who said they were familiar with EHRs in their respective country were able to accurately describe what an EHR is.^21^ Populations need to be able to learn about EHRs, as without education on the matter, it would make it much harder to increase public participation in EHRs.^25^

#### Data transparency

Data transparency refers to the public wanting more clear and concise communication from healthcare system stakeholders on what health data of theirs is being recorded, who is being given access to their records, and what their data is being used for by those who access it. This theme was seen across numerous articles. However, the details of people's specific issues with data transparency were mixed. In one article, the vast majority of participants were unaware that their EHRs were able to be accessed for research purposes.^33^ In other studies, participants were aware their records were used for research, but wanted to know what types of research their data was being used for, and how they were being used to benefit others.^17,29^ Overall, participants mentioned a lack of transparency from the appropriate health system stakeholders, and this led them to be less trusting of EHRs and sometimes even the healthcare system as a whole.^18,26,31^ When patients viewed their EHRs and saw that their physician entered in clear, succinct, and truthful information on the patients’ health status, their trust increased.^34^

#### Operational clarity

Operational clarity relates to the public wanting explicit information on the operation of EHR procedures in healthcare systems, with a particular emphasis on the consent process. For EHRs and depending on the national implementation policy, this consent process is usually referred to as “opt in” and “opt out,” where patients “opt in” when they give consent for their data to be stored on an EHR, or they “opt-out” when they withdraw their consent to have their data be held within an EHR. Across multiple studies, participants found that a lack of clear procedures for opting in or out of having their health data stored in an EHR made them concerned and uneasy about trusting the EHR system.^24,25,30,33^ In one case, this concern was due to a lack of clear “yes” and “no” checkboxes for whether to opt in or out.^24^ In another case, participants found that the process as a whole required “considerable patience” and more in-depth information resources, such as educational materials through the mail or by a “EHR liaison” at their physicians office that can answer any questions and concerns.^25^

### Data protection

One of the constant primary concerns to the general public in relation to EHRs is data protection. Data protection covers the themes of confidentiality, privacy, and security.

#### Confidentiality

Public concern about confidentiality stems from not wanting any identifying information presented on their records except for those who have been authorized to access the records. One aspect of this concern was in terms of research. Many participants are concerned that potentially sensitive health information could be linked to them through certain information that can identify them specifically and remove the protection of anonymity. This could disrupt the confidential nature of their EHRs, which in turn, would cause them to lose trust in the use of EHRs as a repository of their medical information.^17,29^ In addition to these research concerns, it was also discussed that there was worry when medical professionals were recording non-medical information that was subjective opinion or judgment based.^20^ Yet, there still exists some clarity issues on the subject, as there seems to be little consensus among participants on what exactly constitutes identifying information and how their records could be anonymized effectively when required in secondary usage of EHR health data, such as in research.^28^

#### Privacy

Where consensus exists among the public, however, is on the issue of privacy. Participants across multiple studies stated that one of the primary concerns of the public is their data being obtained, whether through lawful or nefarious means, by third party entities that look to exploit a person's health data for their own means.^29,35^ This fear exists in many forms. In some cases, this fear constitutes itself primarily with the concern of hackers accessing their EHRs and using the data found in their records to commit identity fraud or selling the information for profit to other malcontent actors.^23,26,28,36^ Speaking of financial gain, participants also fear commercial companies from being granted access to their records, albeit legally, and using the data contained to sell it for commercial means.^18,26^ This lack of trust also extends to employers and insurance companies. A majority of people are worried about unauthorized access from these groups and are concerned that it can lead to discrimination and unfair judgments in relation to their work or insurance premiums.^23,26,28,37^ This mistrust of third parties gaining access to information for less than scrupulous means is not only relegated to hackers, insurance companies, employers, and commercial companies. Medical professionals are also under the eye of suspicion from the general public. People are nervous of doctors other than the ones involved in their care being able to gain access to the private data stored in their records.^22^ Additionally, people are uneasy about the potential ability of non-clinical healthcare staff to access their EHRs and ascertain their sensitive health information.^37^ Without assurance that their data can remain private and outside the hand of outside groups, trust will remain at a low point and risk to peoples digital health security will stay high, allowing for potential users to remain disinclined in trusting EHRs to contain their health information.^35^

#### Security concerns

In many instances, the public has stated that their chief concern was the security of their records.^21,38,39^ In certain articles, participants suggested solutions to increase data security, measures such as two-factor authentication, or having their records not contained in a national system; however, in most articles, specific information was missing other than stating that people wanted highly secure systems in place and that participants “widespread concern and lack of trust are related to the issues connected with ensuring security…to their medical data.”^19,22,28,29^ These trust issues are in part due to a lack of trust in the data security infrastructure their data might be found in. This seems to differ based on which country the research is being conducted in. Depending on the country, some individuals are confident that their governmental authorities can maintain a high sense of security when it comes to their health records (such as in Sweden), yet, in other countries, (such as the UK), people are fearful that their countries health infrastructure is not up to the task of protecting their health data security.^22,28^ Mistrust in this system also extends to the private sector as well, where nearly all people interviewed in one particular study had low or even very low trust in private companies setting up the database used to store their health records.^19,35^

### Personal benefit

Beginning with the first of two major effect themes, the public views many personal benefits from using EHRs once adequate levels of public trust in them exist. This section will focus on the three major personal benefits the public receive, both implicitly and explicitly.

#### Communication

The public also believes that a further personal benefit of EHRs is the increase in clear communication between a patient and their physician. In the eyes of the public, by having all of their health information stored in one file, it facilitates a more cohesive and comprehensive interaction with their physician. This is because the physician is able to quickly view and analyze all the data on the patient and is able to give faster and more complete direction when it comes to a person's health.^40^ Furthermore, confidence in EHRs was found to be strengthened by the belief that with each subsequent patient–physician encounter, a more detailed, comprehensive, and up-to-date record will be created and made available for both the patient and the physician. This allows for a more constructive and better communicated session between a patient and their physician as they continue to interact.^32^ However, certain individuals are wary of the role of EHRs in facilitating clearer communication. Some people believe that the use of EHRs leads to a more “impersonal” encounter with their doctors, as they seem to focus more on the patients data than on what the patient themselves is relaying.^36^ Yet, in the literature, this seems to be a minority opinion as most interviewed participants across studies suggest that the improved accessibility of complete health-related data brought forth by EHRs gives people and their physicians a sense of seamless and effective communication over time and location.^32,36,40^

#### Empowerment

Empowerment for people comes from explicit and implicit desire from individuals that they want to *feel* more in control of their health information. Even if they end up doing nothing with this authority, by feeling in control of their EHRs, they feel that they are a bigger stakeholder in their health outcomes and that they are empowered over the future of their health.^41,42^ If they lack this empowerment, potential users of EHRs struggle to find a benefit in EHRs and believe there is no necessity to properly maintain and manage the record. If they feel they are the not “the captains steering the ship,” in other words, they feel they are not the ones in control of their health care, they view the use of EHRs as a pointless exercise.^35^

#### Participation

When the public are given access to their health information, they are more motivated to participate in their healthcare and make more of an effort to positively influence their health outcomes. This active participation allows for more personal responsibility in regards to a person's health, which allows people to go from a passive to active participant in the health care process.^42^ In addition to increasing personal responsibility for their health outcomes, this higher involvement in their own care can also prove to be pivotal in improving safety conditions during a medical emergency, leading to better health outcomes during times of health uncertainty.^38^ Patients view this participation as a catalyst to increase the potential of EHRs as an essential tool in the healthcare decision-making process, whether they are making these decisions for themselves or for their loved ones.^27^

### System benefit

When public trust exists in EHRs, in addition to seeing benefits for themselves, much of the public expresses with an altruistic notion the belief that EHRs also can benefit the system and assistance to others within the healthcare system.

#### Efficiency

In multiple studies, participants discussed how the work of physicians can be made simpler due to the ease in which EHRs can bring forth a patients’ important health data. As well, participants believed that the whole of the health system can see a benefit from using EHRs, as, through EHRs, data can be accessed anywhere, at any time.^24,38,40,43^ Yet, many studies did not delve deeper into the subject matter in discussing why patients feel EHRs increase efficiency beyond what was just described. Just one study states that EHRs can lead to an improvement in physicians’ work, legibility, information storage and retrieval, confidentiality, and accuracy, while also reducing the number of times the physician exits from the room.^43^

#### Data sharing in research

The final theme correlated to system benefits is that of data sharing. Data sharing refers to phenomenon that people see data sharing from EHRs, for the most part, in a positive way, in that it can aid those who have the same or similar condition, primarily in terms of research on the matter.^23^ It has been reported that the majority of people support having their records used for research, especially in regards to those who have existing health problems, who are more open to sharing their data than others who do not have such health issues.^17,23^ Furthermore, people want to be able to see how their records benefit others and want to know how they can contribute to new findings in research that can help others like themselves.^29^ On the other hand, while the public is generally supportive of EHR data sharing in research, they still have concerns over the security and privacy of their data. Though, while they are concerned about the security/confidentiality of their data, participants relay that they want their health data to be used for the “common good” if it can contribute to something beneficial.^26,38^

#### Professional networking

The concept of professional networking, which in this context describes the streamlining of communication between physicians and other physicians or medical personnel through EHRs, can reduce rates of miscommunication and therefore medical mistakes. As a secondary effect from enhanced communication between health professionals, people believe as well that with the use of EHRs, patient safety can increase due to an improvement in communication between doctors that is facilitated by the data sharing aspects of EHRs.^38,43^ However, the reviewed articles do not contain in-depth detail beyond these thoughts and feelings.

## Additional findings

Aside of the main findings of our analysis we uncovered three additional findings: firstly, health system actors and their rather significant influence on trust in EHRs; secondly, health data versus financial data security concerns and which takes precedence in the public's view; and thirdly, unique populations that perceive and trust EHRs in a distinctive manner in compared to the general population.

### Role of health system actors for public trust building

**Figure 4.**
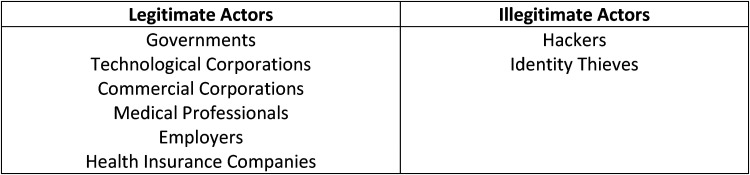
Legitimate and illegitimate health system actors.

Beyond conceptual themes exists a factor that heavily influences trust but is not specifically mentioned in the reviewed literature: Health system actors who are directly involved in EHR development, maintenance, governance, data provision, and data use and illegitimate actors who significantly influence public trust outside of the health system structure. This result came about during the iterative process where the research team discovered and discussed the multiple actors that were seen to play important roles in influencing trust on the general public in the context of EHRs. This result is separated from the key theme due to our belief that actors are not so much their own conceptual theme but are an interconnected thread across the conceptual themes as they are in charge of translating the themes into practice (Figure 4).

These actors are less related to conceptual notions, but are more so related to the human-derived aspects of the trust relationship between the public and EHRs. Depending on their actions and their role in the healthcare system, these actors can have an outsized influence on public trust. For example, private corporations or entities can influence trust, yet usually in a negatively perceived way. Health insurance companies and employers are not that trusted among the public as fears of unauthorized access, discrimination, and higher costs are often associated when these actors are involved in the public trust and EHR relationship.^19,23,26,28,29,35,37^ Additionally, there are public trust concerns with more generalized private, commercial organizations, particularly technological corporations, who partner with national governments to setup and maintain the national EHR system, as well as commercial corporations that profit off of the selling of personal data.^18,19,22,26^ The actors with the least amount of trust, on the other hand, are illegitimate actors who operate outside of the health system with nefarious or malevolent intentions: hackers and identity thieves.^23,26,28^ Government and medical professionals enjoy a more balanced opinion, though depending on the context and country, they can vary between being a positive or negative influence on public trust in EHRs. For governments, effective governing, successful implementation of a national EHR program (or at the very least, being productive and timely on the roll-out of one), and an already existing amount of trust from the public can allow the government to be an effective actor in promoting public trust in EHRs.^21,22,28^ However, even if one of the aforementioned governmental aspects were missing, it can cause governmental intervention to be a liability rather than an asset. In regards to medical professionals, they seemingly have the most profound influence on public trust in an EHR system, though it can be context specific. Medical professionals who were directly involved in a person's care, transparent with patients, received their consent, and communicated well with them had high levels of trust from the public and were highly influential in people having trust in EHRs.^20–22,32,34,43^ Yet, medical professionals can still be actors who damage trust. If certain health professionals gain access to a person's records, such as a physician who was not given consent, a physician not involved in the persons health care, physicians who look at data that is private or redacted, and those who are non-clinical staff, such as administrational professionals, then the trust relationship is damaged between the public and both EHRs and in the health system in general.^20,22,37^

### Public concerns about the security of health data versus financial data

Another finding was that in two studies, the general public seem to be more concerned with the security of their financial data than with their personal health data.^23,31^ While it was not stated whether this is also the case for those with more sensitive health diagnoses, this is nonetheless a notable secondary result. Additionally, Lafky and Horan introduced a security paradox with their findings, where while participants were greatly concerned still for the security of their EHRs, the majority were not inclined to engage in data security activities, especially when there was a financial burden involved. Whether the patients’ health status was “well,” “unwell,” or “disabled,” participants were less willing to engage in a more protective measure if a security device or service had to be purchased (less than 20% of participants indicated they would spend more money if it meant higher security).^23^

### Populations with a distinct attitude towards data sharing

A further discovery is in regard to two segments of the public that have distinct views on data sharing when compared to the general public, but are similar to each other in their views. In one study, it was found that the older and less educated an individual was, the more likely they were willing to share their health data.^27^ Furthermore, it was found that those who were less educated and/or older were also less concerned with the security and confidentiality of their records, compared to those with higher education degrees and/or were younger.^28,30^

## Discussion

Productive and effective health system governance that aims to promote public trust in EHRs relies on a clearer understanding of public trust in EHRs. By reaching a cohesive conception on the matter, a conceptual framework can be developed and shared with policymakers and other stakeholders so that they can be properly guided towards the successful implementation of EHRs into the health system. By amalgamating the current literature on the matter, this scoping review contributes to the field of public trust research and aids healthcare systems in adopting EHRs into their health system through measures that build public trust.

### Common areas of public concern in regards to trust in EHRs

Of the themes presented in the literature, the most commonly cited and addressed concerns of the public when it came to trusting EHRs was the concepts related to data transparency, autonomy, and data protection. The foundation for public trust in EHRs, according to public sentiment, relies upon strengthened transparent practices and operational procedures, strong security measures, and allowing for individuals to be the primary arbiter of their health data.^19,21,22,25,28,29,33^ When the public can trust that their health data is being handled in a secure and responsible manner, that they are informed on how and why their data is being used, and that they feel more in control of their health information, they are more conceivably willing to share it with those involved in their care. This increase in data sharing can improve the quality, coordination, safety, and overall efficiency of the care they receive. However, trust can be difficult to gain and easy to lose. Access from undesired third parties, lack of transparent procedures to withdraw consent (opt-out), loss of autonomy, and low trust in the institutions responsible for the management of EHRs can erode the public's trust.^17,19,21,22,24,26,28,29,35^ Yet, people see the benefits for EHRs, both for themselves and the healthcare system as a whole, and they are generally not opposed to participating in such; but, without their fears and concerns being addressed, they will not partake in EHRs and this could lead to detrimental health outcomes for patients and for the health system.

### Publics views on the need of increased health data security, transparency, and oversight

Across the literature, there were many examples of specific requests that the public had for healthcare stakeholders. Within the literature, participants frequently ask for what they required in order for them to be able to trust EHRs. The most popular of these views is for an opt-in system for EHRs instead of opt-out approach; something that is hard to make feasible and workable, especially when trying to roll out an EHR program on a national scale as gaining consent from a large population (such as on a national scale) can prove to be a heavy burden on the administration of the health system, which can lead to less time focusing on patient care and piloting projects that can benefit patients in other ways.^44^ Some views focus on the information contained within an EHR. For example, some participants felt the that they should be able to negotiate personally with physicians about the specific data points that went into their file, while others took it a step further and expressed a desire to have personal control of redacting certain parts of EHRs themselves.^20,22^ In other cases, public participants had their requirements more focused on research practices. These views include wanting to be personally briefed on what type of research projects their data is being used for, as well as being asked personally for consent beforehand whenever their data would be used for research.^17,29^

By assuaging public concerns for security, transparency, and oversight of health data, it can lead to increased levels of trust in EHRs; as the public is more willing to trust the health system and EHRs with their data if these concerns are addressed.^21,26,31,38,39^ Further research into the relationship between public wants and desires versus what is practical and feasible from a data infrastructure and legislative point of view can lead to potentially interesting results that can assist health policy makers in seeking a junction between realistic policy and public expectations.

### The human element in public trust building

Much of the data within the literature seems to showcase that the public already has at least some level of trust towards EHRs and can see that EHRs bring benefits to both them and to the health system. Yet, why do so many, across multiple studies, have so many issues that cause them to lose trust in the integrity of EHRs? What appears to be the common thread is human involvement. This lack of trust is not from the impersonal EHR or the technology and conceptual framework behind it. Whenever a human actor (such as a hacker) or human based entity (such as an insurance company or governmental institution) gets involved, this is when we see issues of mistrust come in, which only looks to break down the already present trust the public has in EHRs. As mentioned previously in this review, the influential health system actors in this relationship have the power to change how trustful the public is in the use of EHRs, yet many members of the public see a lot of these actors as harmful, or at the very least, capable of doing harm to them through the accessing of their health data; and this capability hinders and capacity for the public to gain trust. This fear in another's capability to do harm stems from a principal presupposition in economics: that “no one knows another's value set.” In the context of this argument, this means that the public is unaware of whether they will be exploited or not, and for what reason is this exploitation occurring.^45^ This fear of the unknown drives distrust in the system as distrust is usually predicated on the uncertainty of what are the future intentions or actions of the other party in the trust relationship.^46^ These other parties, in our context at least, seem to exclusively be other human beings. The issues presented earlier in this paper, transparency, access, control, security, privacy, etc. all stem from the human element, where the common denominator for why these issues are issues are people who conduct themselves in a way that benefits themselves instead of acting benevolently towards the public. Yet, humans can still bring trust back into the relationship instead of chipping it away. The most important individuals in the relationship between public trust and EHRs are physicians, and they carry the most significant weight when it comes to carrying forward public trust in EHRs for the better. This shows that humans are not just destructive in nature when it comes to trust. When we have health system actors who have earned the trust of the public, they can be used to combat the actions of those who create mistrust in our society. Humans, both as agents for and against public trust in EHRs, seem to be the dominant component on this matter. This is because people are involved at every point in the trust relationship, and since human are also a necessity to the deployment of any form of digital system, such as an EHR, it is easy to see how humans are the most significant factor in the relationship between the public and EHRs.^47^ Therefore, as it appears to be that the human element is the most influential factor in determining the level of trust the public has in EHRs, it requires continued study as this can help not only to better understand the intricacies of public trust in EHRs, but how to build trust in any other relationship across society.

### Health versus financial data security and unique populations

Of note, in more than one instance, it was seen that the public is generally more concerned with the security of their financial data than their health data.^23,31^ What was also interesting was that some researchers found that those with health issues were less willing to spend more money on protecting their health data than those that were otherwise healthy. The authors believe that while people report that their health data security is of paramount concern, they are not concerned enough to shoulder any financial burden that would help assuage this concern.^23^ This highlights an attitude that while all personal data should be kept secure, there are select pieces of data that are more important to individuals than others. This suggests that even in the broad context of health data, potentially not all data present on an EHR is equally important to a patient.

Additionally, distinct populations were also found that had a differing view on security and health data sharing than other populations within the public sphere. Those that were older and/or less educated less concerned with their data security and were more willing to share their health data when compared to those who were younger and/or more educated, respectively.^27,28,30^ The authors postulate this can be due to seniors having more information to share, seniors having more implicit trust in their physicians who handle their EHRs, and, for both seniors and the less educated, a desire to seek others who can view their data and help explain and elaborate their own health data.^27,30^ This highlights an interesting viewpoint in public trust of EHRs. The public is nuanced and within the public there are certain populations that act differently than the public as a whole. When aiming to successfully implement EHRs into the healthcare system, healthcare stakeholders must also be aware that certain populations require different approaches when it comes to the public gaining trust in EHRs.

### Existing gaps

Within the literature, prominent gaps in the current knowledge have been identified. First, there were no studies that analyzed the temporal aspect of public trust in EHRs to analyze how the public's trust in EHRs may have evolved over time. Second, there were no studies that went in-depth on trying to understand the conceptualizations behind what public trust in EHRs consists of, leading to a current lack of a clear common conceptual framework in which to analyze public trust in national EHR systems. Third, with the scope of this review only going up until the end of 2021, and COVID-19 and AI having constituted major shakeups in society of the early 2020s, there were no articles that studied the effects of either on the trust relationship between the public and EHRs. Future research on these topics will be of increasing importance and will do much to better understand public trust in EHRs, both now and in the future.

## Limitations

The limitations of this review include the loose definition of “public,” as articles that specifically focused on patient trust in EHRs were included in this review. However, this was done due to the relatively low number of existing literature that focused on public trust in EHRs, and it can be argued that patients are members of the public, with the key difference being that patients are actively being treated within the health system, as opposed to the greater public who are not. As well, also due to the low abundance of public trust in EHR literature, the search string for finding the articles included in this review was very generalized. However, this generalized search allowed us to widen our search and discover articles that may not have been found with a more specific search string.

## Conclusion

EHRs are known to be a great resource in both healthcare and public health research and surveillance. However, there remain obstacles to their implementation in multiple settings around the world. Many of these obstacles trace their origin to a problem in public trust. Without the existence of public trust in the security, privacy, and righteousness of their personal health data in EHRs, public support and participation will be nonexistent, therefore rendering the implementation of EHRs as a failure. Furthermore, it is complicated by the fact that increasing the trust the public has in the humans that operate and influence the EHR system is no easy task in of itself; as this trust is not easy to gain or maintain, but it can be easily lost. Yet, despite these hurdles, we can continue to make progress in researching the conceptual frameworks surrounding the relationship between public trust and EHRs. With our findings, we contribute to a better conceptual understanding of what public trust in EHRs is so that health system professionals and policy makers can better implement EHRs in their healthcare system.

## Supplemental Material

sj-pdf-1-dhj-10.1177_20552076241228024 - Supplemental material for What is public trust in national electronic health record systems? A scoping review of qualitative research studies from 1995 to 2021 Click here for additional data file. Supplemental material, sj-pdf-1-dhj-10.1177_20552076241228024 for What is public trust in national electronic health record systems? A scoping review of qualitative research studies from 1995 to 2021 by Kimon Papadopoulos, Viktor von Wyl and Felix Gille in DIGITAL HEALTH

sj-xlsx-2-dhj-10.1177_20552076241228024 - Supplemental material for What is public trust in national electronic health record systems? A scoping review of qualitative research studies from 1995 to 2021 Click here for additional data file. Supplemental material, sj-xlsx-2-dhj-10.1177_20552076241228024 for What is public trust in national electronic health record systems? A scoping review of qualitative research studies from 1995 to 2021 by Kimon Papadopoulos, Viktor von Wyl and Felix Gille in DIGITAL HEALTH

sj-docx-3-dhj-10.1177_20552076241228024 - Supplemental material for What is public trust in national electronic health record systems? A scoping review of qualitative research studies from 1995 to 2021 Click here for additional data file. Supplemental material, sj-docx-3-dhj-10.1177_20552076241228024 for What is public trust in national electronic health record systems? A scoping review of qualitative research studies from 1995 to 2021 by Kimon Papadopoulos, Viktor von Wyl and Felix Gille in DIGITAL HEALTH

sj-jpeg-4-dhj-10.1177_20552076241228024 - Supplemental material for What is public trust in national electronic health record systems? A scoping review of qualitative research studies from 1995 to 2021 Click here for additional data file. Supplemental material, sj-jpeg-4-dhj-10.1177_20552076241228024 for What is public trust in national electronic health record systems? A scoping review of qualitative research studies from 1995 to 2021 by Kimon Papadopoulos, Viktor von Wyl and Felix Gille in DIGITAL HEALTH

## Review Articles

Alaqra AS, Fischer-Hübner S, Framner E. Enhancing privacy controls for patients via a selective authentic electronic health record exchange service: qualitative study of perspectives by medical professionals and patients. *J Med Internet Res* 2018; 20: 13–13.

Asan O, Tyszka J, Fletcher KE. Capturing the patients’ voices: planning for patient-centered electronic health record use. *Int J Med Inf* 2016; 95: 1–7.

Botkin JR, Rothwell E, Anderson R, et al. Public attitudes regarding the use of electronic health information and residual clinical tissues for research. *J Community Genet* 2014; 5: 205–213.

Cajander Å, Grünloh C. Electronic health records are more than a work tool: conflicting needs of direct and indirect stakeholders. In: *Proceedings of the 2019 CHI Conference on Human Factors in Computing Systems*. Glasgow Scotland Uk: ACM, pp.1–13.

Carman D, Britten N. Confidentiality of medical records: the patient's perspective. *Br J Gen Pract*.

Clerkin P, Buckley BS, Murphy AW, et al. Patients’ views about the use of their personal information from general practice medical records in health research: a qualitative study in Ireland. *Fam Pract* 2013; 30: 105–112.

Cromer R, Denneson LM, Pisciotta M, et al. Trust in mental health clinicians among patients who access clinical notes online. *Psychiatr Serv Wash DC* 2017; 68: 520–523.

Damschroder LJ, Pritts JL, Neblo MA, et al. Patients, privacy and trust: patients’ willingness to allow researchers to access their medical records. *Soc Sci Med* 2007; 64: 223–235.

Hammack-Aviran CM, Brelsford KM, McKenna KC, et al. Research use of electronic health records: patients’ views on alternative approaches to permission. *AJOB Empir Bioeth* 2020; 11: 172–186.

Hoerbst A, Kohl CD, Knaup P, et al. Attitudes and behaviors related to the introduction of electronic health records among Austrian and German citizens. *Int J Med Inf* 2010; 79: 81–89.

Lafky DB, Horan TA. Personal health records: consumer attitudes toward privacy and security of their personal health information. *Health Informatics J* 2011; 17: 63–71.

Lounsbury O, Roberts L, Goodman JR, et al. Opening a “can of worms” to explore the public's hopes and fears about health care data sharing: qualitative study. *J Med Internet Res* 2021; 23: e22744.

Maiorana A, Steward WT, Koester KA, et al. Trust, confidentiality, and the acceptability of sharing HIV-related patient data: lessons learned from a mixed methods study about Health Information Exchanges. *Implement Sci IS* 2012; 7: 34.

Morin D, Tourigny A, Pelletier D, et al. Seniors’ views on the use of electronic health records. *Inform Prim Care* 2005; 13: 125–133.

Nurgalieva L, Cajander Å, Moll J, et al. ‘I do not share it with others. No, it's for me, it's my care’: on sharing of patient accessible electronic health records. *Health Informatics J* 2020; 26: 2554–2567.

Papoutsi C, Reed JE, Marston C, et al. Patient and public views about the security and privacy of Electronic Health Records (EHRs) in the UK: results from a mixed methods study. *BMC Med Inform Decis Mak* 2015; 15: 86.

Richter JG, Becker A, Koch T, et al. Changing attitudes towards online electronic health records and online patient documentation in rheumatology outpatients. *Clin Exp Rheumatol* 2010; 28: 261–264.

Shield RR, Goldman RE, Anthony DA, et al. Gradual electronic health record implementation: new insights on physician and patient adaptation. *Ann Fam Med* 2010; 8: 316–326.

Simon SR, Evans JS, Benjamin A, et al. Patients’ attitudes toward electronic health information exchange: qualitative study. *J Med Internet Res* 2009; 11: e30.

Söderström E, Eriksson N, Åhlfeldt R-M. Managing healthcare information: analyzing trust. *Int J Health Care Qual Assur* 2016; 29: 786–800.

Spencer K, Sanders C, Whitley EA, et al. Patient perspectives on sharing anonymized personal health data using a digital system for dynamic consent and research feedback: a qualitative study. *J Med Internet Res* 2016; 18: e66.

Spil T, Klein R. The personal health future. *Health Policy Technol* 2015; 4: 131–136.

Stablein T, Hall JL, Pervis C, et al. Negotiating stigma in health care: disclosure and the role of electronic health records. *Health Sociol Rev* 2015; 24: 227–241.

Sterckx S, Rakic V, Cockbain J, et al. “You hoped we would sleep walk into accepting the collection of our data”: controversies surrounding the UK care.data scheme and their wider relevance for biomedical research. *Med Health Care Philos* 2016; 19: 177–190.

Stevenson F, Lloyd N, Harrington L, et al. Use of electronic patient records for research: views of patients and staff in general practice. *Fam Pract* 2013; 30: 227–232.

Ventres W. Physicians, patients, and the electronic health record: an ethnographic analysis. *Ann Fam Med* 2006; 4: 124–131.

Wiśniewska J, Różycka M. The problem of trust in innovative ICT technologies used in e-health systems. The case study of private health care units located in Szczecin. *Knowl-Based Intell Inf Eng Syst Proc 25th Int Conf KES2021* 2021; 192: 3647–3656.
