# Supplementary material for: What is public trust in national electronic health record systems? A scoping review of qualitative research studies from 1995 to 2021
Source: Digit Health. 2024 Jan 28;10:20552076241228024. doi: 10.1177/20552076241228024 (PMC10823845; doi:10.1177/20552076241228024)
Supplement: sj-docx-3-dhj-10.1177_20552076241228024 - Supplemental material for What is public trust in national electronic health record systems? A scoping review of qualitative research studies from 1995 to 2021 [file sj-docx-3-dhj-10.1177_20552076241228024.docx]

| MAXQDA Codes | | |
| --- | --- | --- |
| Communication | Efficiency | Participation |
| eHealth | Awareness | Transparency |
| Empowerment | Clarity | Autonomy |
| Permission | Anonymity | Security |
| Control | Access | Health Records |
| Data Protection | Consent | Data Sharing |
| Confidentiality | Trust | Privacy |
