# Supplementary figures and images for: What is public trust in national electronic health record systems? A scoping review of qualitative research studies from 1995 to 2021
Source: Digit Health. 2024 Jan 28;10:20552076241228024. doi: 10.1177/20552076241228024 (PMC10823845; doi:10.1177/20552076241228024)

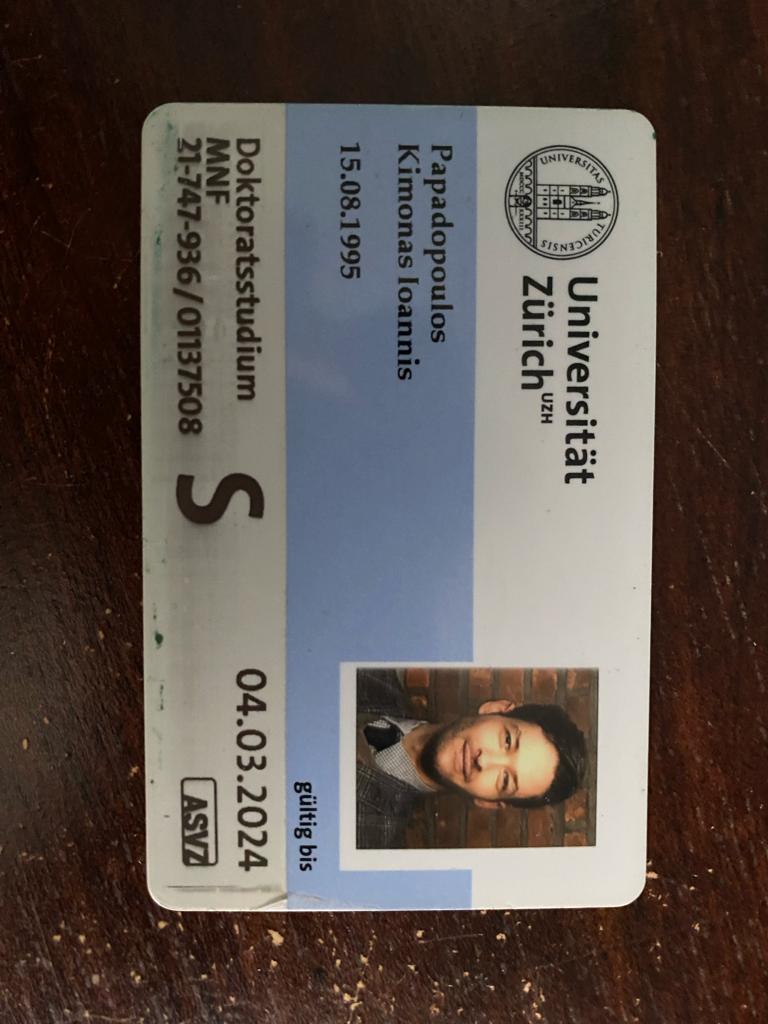

Supplement: sj-jpeg-4-dhj-10.1177_20552076241228024 - Supplemental material for What is public trust in national electronic health record systems? A scoping review of qualitative research studies from 1995 to 2021 [file sj-jpeg-4-dhj-10.1177_20552076241228024.jpeg]
